# Activin B induces human endometrial cancer cell adhesion, migration and invasion by up-regulating integrin β3 via SMAD2/3 signaling

**DOI:** 10.18632/oncotarget.5229

**Published:** 2015-08-28

**Authors:** Siyuan Xiong, Christian Klausen, Jung-Chien Cheng, Hua Zhu, Peter C.K. Leung

**Affiliations:** ^1^ Department of Obstetrics and Gynaecology, Child and Family Research Institute, University of British Columbia, Vancouver, British Columbia, Canada

**Keywords:** activin B, inhibin subunit βB, integrin β3, integrin αv, serous endometrial cancer

## Abstract

Endometrial cancer is the fourth most common female cancer and the most common gynecological malignancy. Although it comprises only ~10% of all endometrial cancers, the serous histological subtype accounts for ~40% of deaths due to its aggressive behavior and propensity to metastasize. Histopathological studies suggest that elevated expression of activin/inhibin βB subunit is associated with reduced survival in non-endometrioid endometrial cancers (type II, mostly serous). However, little is known about the specific roles and mechanisms of activin (βB dimer) in serous endometrial cancer growth and progression. In the present study, we examined the biological functions of activin B in type II endometrial cancer cell lines, HEC-1B and KLE. Our results demonstrate that treatment with activin B increases cell migration, invasion and adhesion to vitronectin, but does not affect cell viability. Moreover, we show that activin B treatment increases integrin β3 mRNA and protein levels via SMAD2/3-SMAD4 signaling. Importantly, siRNA knockdown studies revealed that integrin β3 is required for basal and activin B-induced cell migration, invasion and adhesion. Our results suggest that activin B-SMAD2/3-integrin β3 signaling could contribute to poor patient survival by promoting the invasion and/or metastasis of type II endometrial cancers.

## INTRODUCTION

Endometrial cancer is the most common, and second most lethal, gynecological malignancy and the fourth most common female cancer in North America [1]. Traditionally, endometrial cancers have been broadly classified into two clinicopathological types [2]. Accounting for ~70% of endometrial cancers, type I tumors are primarily comprised of low-grade endometrioid carcinomas associated with unopposed estrogen and favorable prognosis. In contrast, type II endometrial cancers are predominantly non-endometrioid (serous and clear cell) carcinomas associated with advanced stage and poor survival [3, 4]. In particular, despite accounting for only ~10% of all endometrial cancers, serous endometrial carcinomas account for ~40% of deaths due to their high grade, deep myometrial invasion and propensity for extrauterine spread [3, 5]. In The Cancer Genome Atlas's recent genomic characterization of endometrial carcinomas, serous tumors and ~25% of high-grade endometrioid tumors were grouped in a novel genomic class (copy-number high, serous-like) characterized by extensive copy number alterations, frequent TP53 mutations, and poor outcome [6].

Activins are disulfide-linked homodimers of inhibin β subunits which belong to the transforming growth factor-beta (TGF-β) superfamily [7]. The primary isoforms of activins are activin A (βAβA), activin AB (βAβB) and activin B (βBβB). Activins are expressed in many reproductive tissues, including the endometrium, where they regulate numerous biological functions in an autocrine/paracrine manner [8]. In humans, transcripts encoding inhibin βA and βB subunits as well as activin receptors have been detected in primary cultures of normal endometrial epithelial and stromal cells [9]. At the protein level, secreted activin A has been detected in conditioned medium from normal endometrial epithelial and stromal cells [9], and immunohistochemical analyses have confirmed endometrial expression of inhibin βA and βB subunits throughout the human menstrual cycle [10].

Additionally, increasing evidence suggests that activins and their receptors may participate, either positively or negatively, in the development or progression of a variety of endocrine-related cancers [11]. In endometrial cancer, early studies demonstrated inhibin β subunit expression, activin secretion and activin receptor expression in neoplastic endometrial tissues and/or endometrial cancer cell lines [9, 12, 13]. Histopathological studies have since examined the expression of inhibin βA and βB subunits in sizeable cohorts of endometrial carcinomas of either endometrioid [14, 15] or non-endometrioid [16] histology. In endometrioid tumors, positive immunostaining for inhibin βA or βB was correlated with higher grade, though neither subunit was associated with overall, progression free or cause specific survival [14, 15]. In contrast, non-endometrioid tumors (~70% serous) more frequently displayed positive immunostaining for inhibin βA or βB [15]; however, only elevated inhibin βB was associated with reduced cause specific survival [16]. Interestingly, double immunofluorescence staining of endometrioid tumors showed marked co-localization of inhibin α and βA subunits (suggesting production of inhibin A), whereas there was minimal co-localization of inhibin α and βB, suggesting production of activin B [17].

Though histopathological studies suggest activin B may be linked to poor survival in the most lethal subtype of endometrial cancer, few studies have examined the effects of activins on endometrial cancer cells, and all have examined only the effects of activin A. Early studies demonstrated both pro- and anti-proliferative effects of activin A on HEC-50 and ISH endometrial cancer cells, respectively [13]. However, subsequent studies with HEC-1, HHUA and Ishikawa endometrial cancer cells failed to show any effects of activin A on cell proliferation [12, 18]. In the present study, we examined the effects of activin B on endometrial cancer cell proliferation, migration, invasion and adhesion. We show that activin B does not affect the viability of HEC-1B and KLE type II endometrial cancer cells. However, our results reveal an important role for activin B in promoting HEC-1B and KLE cell migration, invasion and adhesion to vitronectin. In addition, we show that the effects of activin B on cell migration, invasion and adhesion to vitronectin are mediated by the SMAD2/3-SMAD4-dependent up-regulation of integrin β3. Our findings suggest that activin B signaling could promote the invasion and/or metastasis of type II endometrial cancers, thereby contributing to poor patient survival.

## RESULTS

### Activin B increases endometrial cancer cell migration, invasion and adhesion

In a previous study of 41 non-endometrioid tumors (29 serous, 7 clear cell and 5 undifferentiated), positive immunostaining for inhibin βB was observed in approximately half of the cases and was associated with reduced cause specific survival (*P* = 0.026) and trends towards reduced progression free (*P* = 0.111) and overall (*P* = 0.166) survival [16]. Similarly, Kaplan-Meier analysis of endometrial cancers with serous histology from The Cancer Genome Atlas (TCGA) ([6]; *n* = 53) shows that samples with inhibin βB mRNA levels greater than the median are associated with reduced disease free survival (Log-rank *P* = 0.021, Supplementary Figure 1A) and a trend towards reduced overall survival (Log-rank *P* = 0.094, Supplementary Figure 1B). Together, these studies suggest that activin B (βB dimer) signaling could contribute to poor survival in type II serous endometrial cancer.

**Figure 1:**
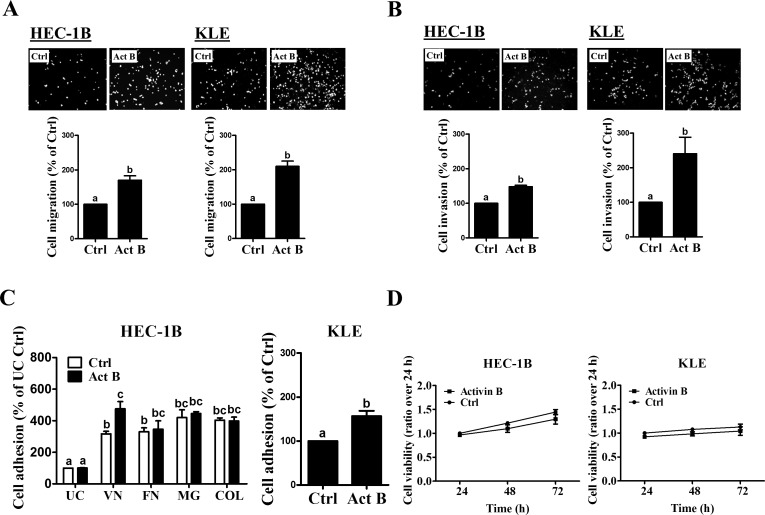
Activin B increases endometrial cancer cell migration, invasion and adhesion A and B, HEC-1B and KLE cells were treated without (Ctrl) or with 50 ng/mL activin B (Act B) for 24 h and then seeded in un-coated **A.** or Matrigel-coated **B.** transwell inserts for migration or invasion assays, respectively. *Upper panels* show representative photomicrographs of migrating/invading cells, while *lower panels* show summarized quantitative results. **C.**, HEC-1B cells were treated with 50 ng/mL activin B for 24 h and then subjected to adhesion assays in un-coated (UC) plates or plates coated with vitronectin (VN), fibronectin (FN), Matrigel (MG) or collagen IV (COL). Additionally, adhesion assays were performed in un-coated or vitronectin-coated plates following treatment of KLE cells with 50 ng/mL activin B for 24 h. **D.**, HEC-1B and KLE cells were treated with 50 ng/mL activin B every 24 h for up to 72 h and cell viability was examined by MTT assay. Results are expressed as the mean ± SEM of at least three independent experiments. Values without a common letter are significantly different (*P* < 0.05).

Next, we examined the biological functions of activin B in two type II endometrial cancer cell lines (HEC-1B and KLE). Transwell migration and Matrigel invasion assay results showed that both HEC-1B and KLE cells exhibited basal levels of cell motility and invasiveness (Figures 1A and 1B). Importantly, treatment with 50 ng/mL activin B significantly increased cell migration and invasion in both cell lines (Figures 1A and 1B). In addition, we examined the effects of activin B on cell adhesion to different extracellular matrix proteins. As shown in Figure 1C, HEC-1B cell adhesiveness was increased in vitronectin-, fibronectin-, Matrigel- or collagen IV-coated tissue culture plates compared to uncoated plates. Interestingly, treatment with 50 ng/mL activin B significantly enhanced the adhesion of HEC-1B cells to vitronectin, but did not affect adhesion to the other extracellular matrix proteins or uncoated plates (Figure 1C). Similarly, activin B treatment increased the adhesion of KLE cells to vitronectin (Figure 1C). MTT assay was used to investigate if the effects of activin B on HEC-1B and KLE cell migration, invasion and adhesion could result from changes in cell viability/proliferation. As shown in Figure 1D, treatment with 50 ng/mL activin B every 24 h for up to 72 h did not affect HEC-1B or KLE cell viability.

**Figure 2:**
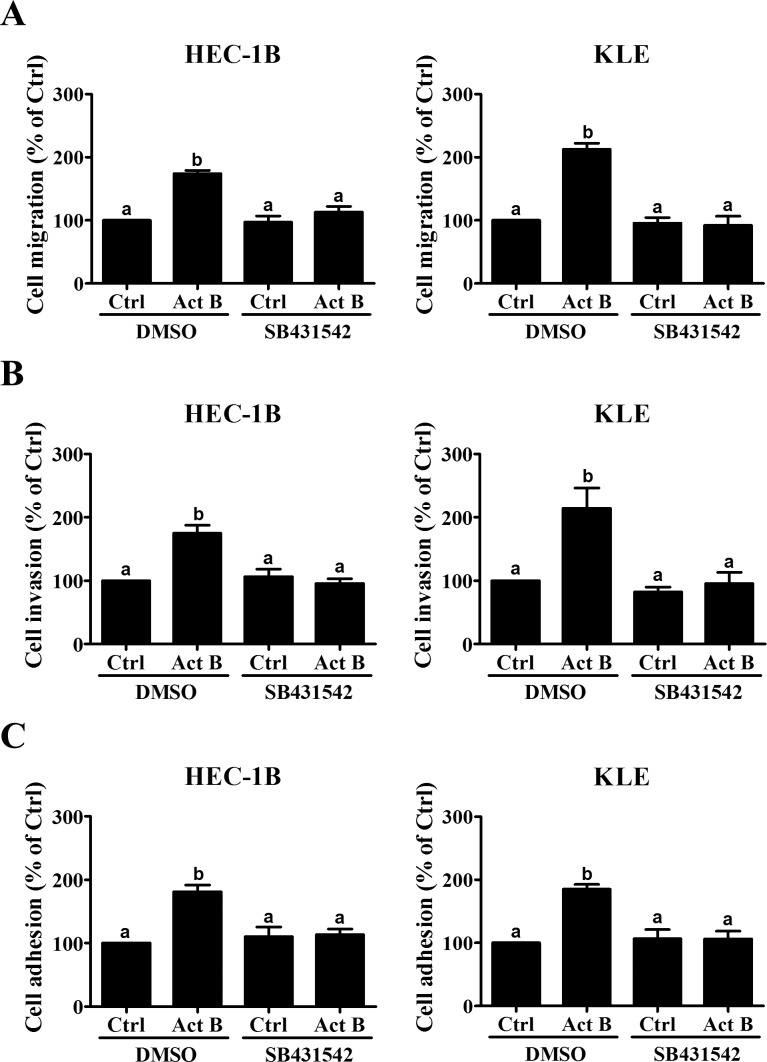
SB431542 abolishes activin B-induced cell migration, invasion and adhesion Migration **A.**, invasion **B.** and vitronectin adhesion **C.** assays were performed with HEC-1B and KLE cells following pre-treatment with vehicle control (DMSO) or SB431542 (10 μM) for 1 h prior to treatment without (Ctrl) or with 50 ng/mL activin B (Act B) for a further 24 h. Results are expressed as the mean ± SEM of at least three independent experiments. Values without a common letter are significantly different (*P* < 0.05).

We also pre-treated HEC-1B and KLE cells with the inhibitor SB431542 to determine whether activin/TGF-β type I receptors were required for the biological functions of activin B. As shown in Figure 2, pre-treatment with SB431542 completely abolished the effects of activin B on cell migration, invasion and adhesion to vitronectin.

### Activin B up-regulates integrin β3 but not integrin αv

**Figure 3:**
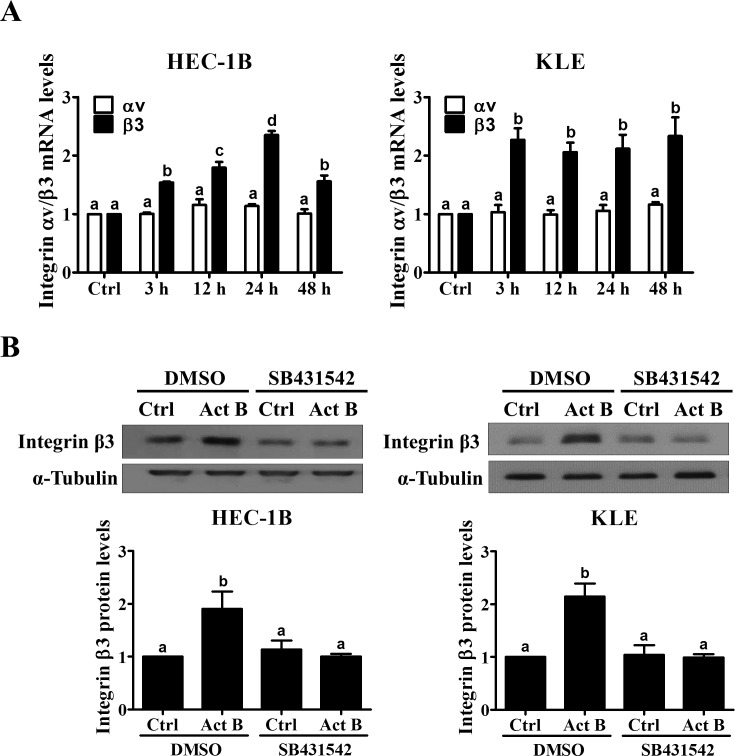
Activin B up-regulates integrin β3 expression in endometrial cancer cells **A.**, HEC-1B and KLE cells were treated for varying times without (Ctrl; time-matched controls displayed as single bar) or with 50 ng/mL activin B (Act B) and integrin αv and β3 mRNA levels were measured by RT-qPCR. **B.**, HEC-1B and KLE cells were pre-treated with vehicle control (DMSO) or SB431542 (10 μM) for 1 h and then treated with 50 ng/mL activin B for 24 h. Protein levels of integrin β3 were examined by Western blot (quantified data are normalized to α-tubulin) ting. Results are expressed as the mean ± SEM of at least three independent experiments. Values without a common letter are significantly different (*P* < 0.05).

Given that activin B specifically enhanced endometrial cancer cell adhesion to vitronectin, we next examined its effects on the levels of integrin αvβ3, well known to be a major receptor for vitronectin [19]. As shown in Figure 3A, treatment with activin B for different periods of time did not affect the mRNA levels of integrin αv in HEC-1B or KLE cells. However, activin B treatment for 3 h significantly up-regulated integrin β3 mRNA levels and this effect was still observed after 48 h of treatment (Figure 3A). Western blot analysis was used to confirm the similar stimulatory effects of activin B on integrin β3 protein levels, and to show that they could be abolished by pre-treatment with SB431542 (Figure 3B).

### SMAD2/3-SMAD4 signaling is required for the up-regulation of integrin β3 by activin B

**Figure 4:**
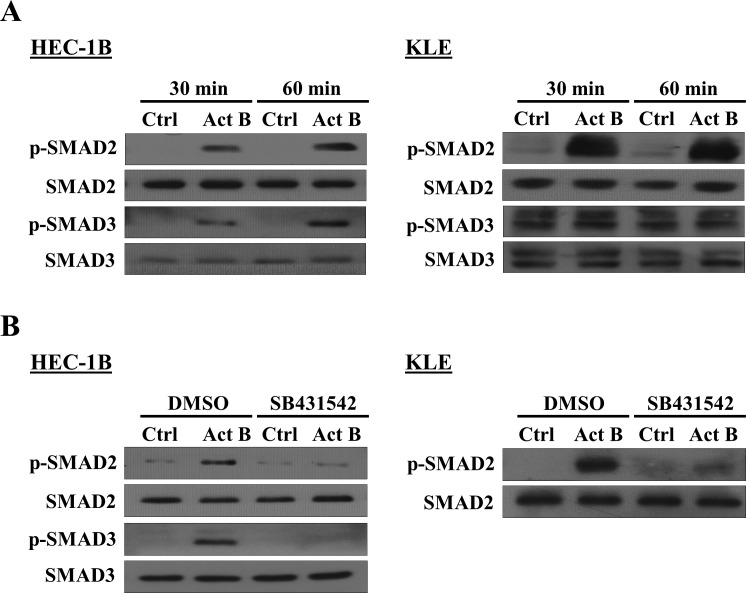
Effects of activin B on SMAD2 and SMAD3 phosphorylation in endometrial cancer cells **A.**, HEC-1B and KLE cells were treated without (Ctrl) or with 50 ng/mL activin B (Act B) for 30 or 60 min and Western blot was used to measure the levels of phosphorylated SMAD2 (p-SMAD2) and SMAD3 (p-SMAD3) in relation to their total levels (SMAD2 and SMAD3, respectively). **B.**, HEC-1B and KLE cells were pre-treated with vehicle control (DMSO) or SB431542 (10 μM) for 1 h and then treated with 50 ng/mL activin B for 60 min. SMAD2 and SMAD3 phosphorylation was examined by Western blot.

To examine the activation of canonical SMAD2/SMAD3 signaling, HEC-1B and KLE cells were treated with activin B and Western blot was used to measure the levels of phosphorylated SMAD2 and SMAD3 in relation to their total levels. As shown in Figure 4A, treatment with activin B for 30 or 60 min induced the phosphorylation of SMAD2 and SMAD3 in HEC-1B cells, whereas only SMAD2 phosphorylation was increased in KLE cells. Moreover, activin B-induced phosphorylation of SMAD2 and SMAD3 in HEC-1B cells as well as SMAD2 in KLE cells was blocked by pre-treatment with SB431542 (Figure 4B).

**Figure 5:**
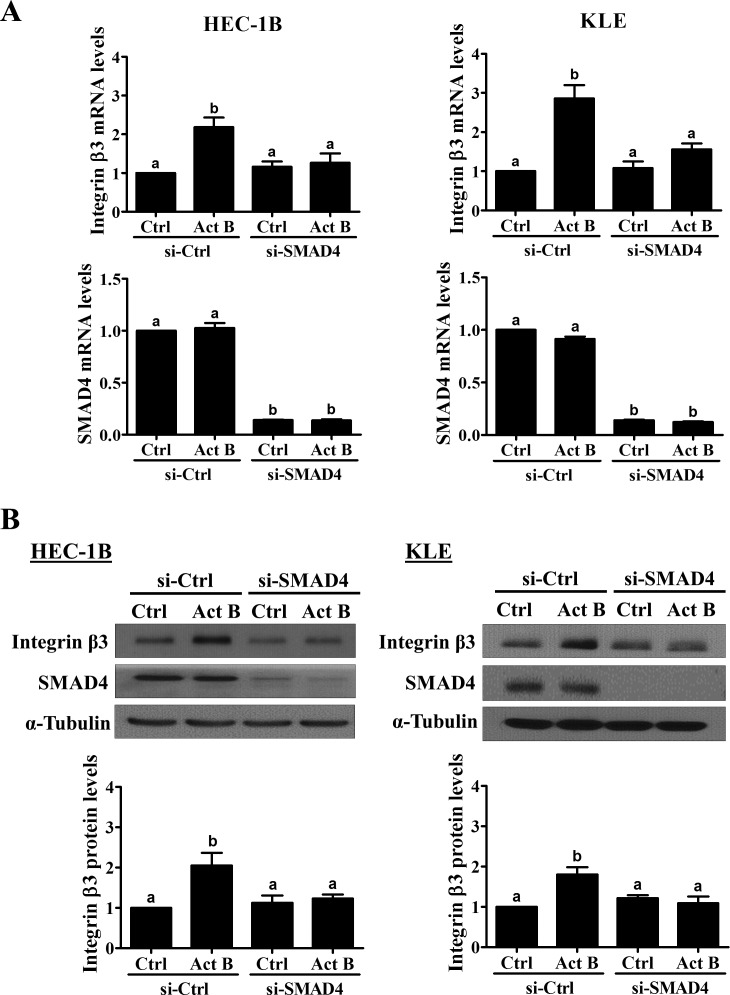
SMAD4 is required for the up-regulation of integrin β3 by activin B HEC-1B and KLE cells were transfected for 48 h with 20 nM control siRNA (si-Ctrl) or SMAD4 siRNA (si-SMAD4) and then treated without (Ctrl) or with 50 ng/mL activin B (Act B) for 24 h. Integrin β3 and SMAD4 mRNA **A.** and protein **B.** levels were measured by RT-qPCR and Western blot, respectively. Results are expressed as the mean ± SEM of at least three independent experiments. Values without a common letter are significantly different (*P* < 0.05).

Next, we used pre-treatment with siRNA targeting common SMAD4 to investigate the involvement of SMAD signaling in the up-regulation of integrin β3 by activin B. As shown in Figure 5A, transfection with SMAD4 siRNA significantly reduced endogenous SMAD4 mRNA levels and abolished the up-regulation of integrin β3 mRNA by activin B in both HEC-1B and KLE cells. Similarly, Western blot analysis showed that activin B-induced increases in integrin β3 protein levels were abolished by pre-treatment of HEC-1B and KLE cells with SMAD4 siRNA (Figure 5B).

**Figure 6:**
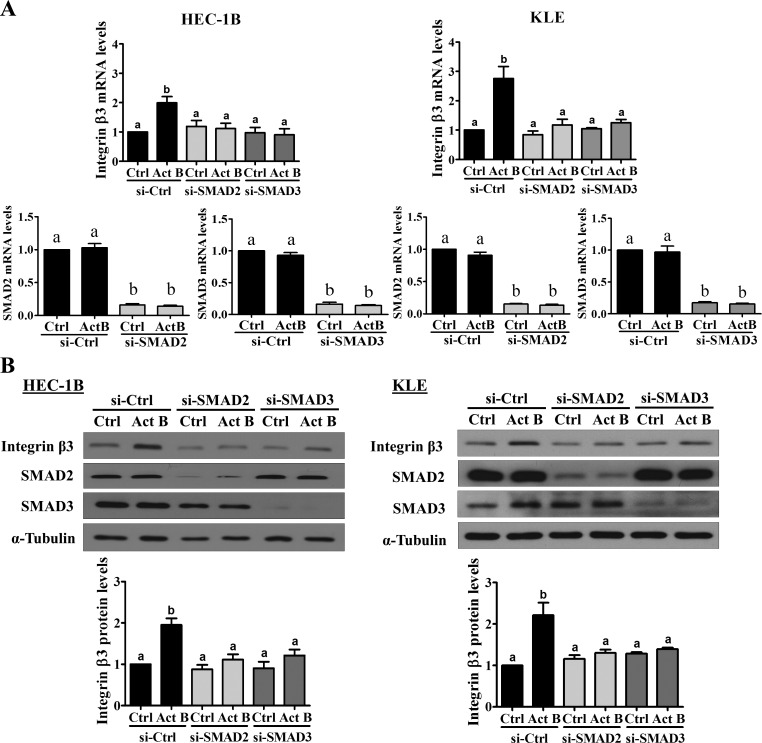
SMAD2 and SMAD3 are required for the up-regulation of integrin β3 by activin B HEC-1B and KLE cells were transfected for 48 h with 20 nM control siRNA (si-Ctrl), SMAD2 siRNA (si-SMAD2) or SMAD3 siRNA (si-SMAD3) and then treated without (Ctrl) or with 50 ng/mL activin B (Act B) for 24 h. Integrin β3, SMAD2 and SMAD3 mRNA **A.** and protein **B.** levels were measured by RT-qPCR and Western blot, respectively. Results are expressed as the mean ± SEM of at least three independent experiments. Values without a common letter are significantly different (*P* < 0.05).

SMAD2 and SMAD3 have been shown to mediate TGF-β-regulated gene expression both redundantly and differentially depending on the cellular context [20]. Therefore, specific siRNAs targeting SMAD2 or SMAD3 were used to investigate their individual roles in the effects of activin B on integrin β3 expression in HEC-1B cells. As shown in Figure 6A, transfection with siRNA targeting SMAD2 or SMAD3 significantly reduced their respective mRNA levels and abolished the effects of activin B on integrin β3 mRNA. Likewise, Western blot analysis showed that activin B-induced increases in integrin β3 protein levels were abolished by pre-treatment of HEC-1B cells with siRNAs targeting SMAD2 or SMAD3 (Figure 6B). Interestingly, though activin B only increased SMAD2 phosphorylation in KLE cells, its stimulatory effects on integrin β3 mRNA and protein levels were abolished following pre-treatment of KLE cells with siRNA targeting SMAD2 or SMAD3 (Figure 6).

### Integrin β3 mediates activin B-induced cell migration, invasion and adhesion to vitronectin

**Figure 7:**
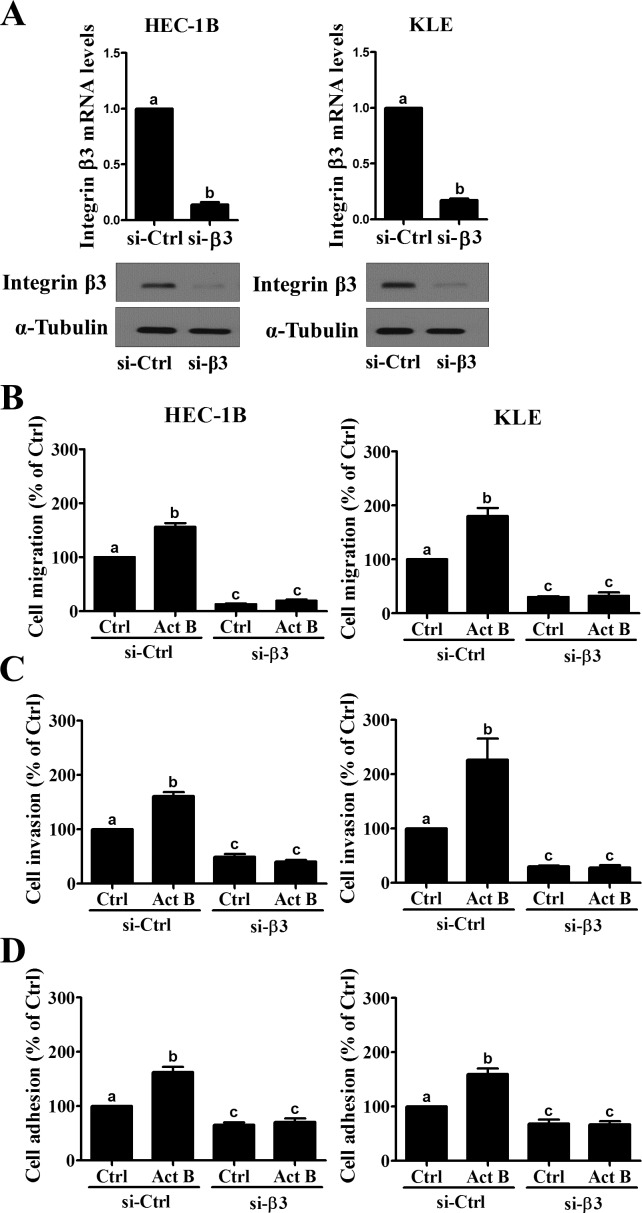
Integrin β3 mediates activin B-induced endometrial cancer cell migration, invasion and adhesion to vitronectin **A.**, HEC-1B and KLE cells were transfected for 48 h with 20 nM control siRNA (si-Ctrl) or integrin β3 siRNA (si-β3) and knockdown efficiencies were examined by RT-qPCR and Western blot. Migration **B.**, invasion **C.** and vitronectin adhesion **D.** assays were performed with HEC-1B and KLE cells following transfection with 20 nM control or integrin β3 siRNA for 48 h prior to treatment without (Ctrl) or with 50 ng/mL activin B (Act B) for a further 24 h. Results are expressed as the mean ± SEM of at least three independent experiments. Values without a common letter are significantly different (*P* < 0.05).

Pre-treatment with siRNA targeting integrin β3 was used to investigate its role in activin B-induced cell migration, invasion and adhesion to vitronectin. As shown in Figure 7A, transfection with integrin β3 siRNA significantly down-regulated integrin β3 mRNA and protein levels in both HEC-1B and KLE cells. Transwell migration and Matrigel invasion assays showed that integrin β3 knockdown suppressed the motility and invasiveness of both HEC-1B and KLE cells (Figures 7B and 7C). In addition, activin B-induced cell migration and invasion were abolished by pre-treatment with integrin β3 siRNA (Figures 7B and 7C). Similarly, integrin β3 knockdown reduced both basal and activin B-induced cell adhesion to vitronectin in HEC-1B and KLE cells (Figure 7D).

## DISCUSSION

Previous studies have demonstrated differences between activin A and activin B with respect to expression patterns, receptor/antagonist binding affinities, and biological functions [21-26]. Indeed, inhibin βA (*Inhba*) and βB (*Inhbb*) subunit knockout mice exhibit different phenotypes, and the defects observed in *Inhba* knockout mice are only partially restored by insertion of *Inhbb* [27, 28]. On the other hand, we have recently demonstrated that recombinant activin A, B and AB have similar effects on human ovarian granulosa cell steroidogenesis and placental trophoblast cell invasion [29, 30]. Taken together, these studies suggest that activin A and B could function distinctly or similarly depending on the cellular context. Our results showing that activin B does not affect endometrial cancer cell viability are in agreement with previous studies examining the proliferative effects of activin A [12, 18]. However, we cannot definitively rule out potential effects of activin B on cell proliferation due to the low proliferative rates of HEC-1B and KLE cells and the limited time-course of our studies. Indeed, activin A has been shown to inhibit the proliferation of estrogen-responsive ISH endometrial cancer cells, whereas it enhanced the proliferation of estrogen-insensitive HEC-50 endometrial cancer cells [13]. Interestingly, treatment with estradiol abolished the suppressive effects of activin A on ISH cell proliferation, whereas it had no impact on the effects of activin A in HEC50 cells [13]. These results suggest that the effects of activins could be modified by other hormones or paracrine factors, perhaps differentially depending on the activin isoform or type I *vs.* type II carcinomas. In renal cancer cells, treatment with activin B had no effect on proliferation *in vitro*, however inhibin βB subunit knockdown cells formed smaller tumors in xenograft studies [31]. Conversely, activins could differentially modulate the effects of other hormones or paracrine factors. For example, activin A has been shown to reduce the growth inhibitory effects of TGF-β on endometrial cancer cell proliferation [18]. Future studies will be required to fully characterize the specific roles and molecular determinants of activin A and B in endometrial cancer cell proliferation.

Commonly confined to the uterus, type I endometrial cancers can often be treated by hysterectomy and have good prognosis. In contrast, the most prevalent type II endometrial cancer, serous endometrial carcinoma, has a relapse rate as high as 50% [32] and accounts for 40% of all endometrial cancer related deaths [5]. This lethality is largely due to its propensity for deep invasion and metastatic spread, thus understanding the molecular mechanisms mediating these processes could lead to new therapeutic approaches for type II endometrial cancers. We now demonstrate, for the first time, that treatment with activin B increases type II endometrial cancer cell migration, invasion and adhesion to vitronectin. To date, only a few studies have examined the effects of activin B on cancer cell motility or adhesion. In renal cancer cells, activin B has been shown to increase cell adhesion and invasion [31, 33], however the molecular mechanisms underlying these effects remain unclear. Together with previous histopathological studies, our results suggest that antagonizing activin B signaling could be a novel approach to the treatment of type II endometrial cancer. However, only a few therapeutic agents targeting the activin system have entered clinical trials (www.clinicaltrials.gov), and none of them are entirely specific for activin signaling [34]. LY-217299 (Galunisertib) is a small-molecule inhibitor of activin/TGF-β type I receptors (related to SB431542) which is currently under phase 1/2 investigation in hepatocellular, pancreatic and glial cancers. Sotatercept (ACE-011) is an activin receptor type IIA (ACVR2A) Fc fusion protein which is currently under phase 1/2 investigation for the treatment of cancer-associated anemia. Interestingly, an ACVR2B Fc fusion protein (STM 434) has entered phase 1 studies in combination with liposomal doxorubicin in patients with ovarian cancer or other advanced tumors, including endometrial cancer (NCT02262455).

Our results show that treatment of endometrial cancer cells with activin B up-regulates integrin β3 mRNA and protein levels without altering the expression of integrin αv. Integrin β3 forms only two α-β heterodimers, αvβ3 and αIIbβ3, both of which are receptors for vitronectin [35]. However, integrin αIIb mRNA levels are nearly undetectable in HEC-1B and KLE cells, whereas the mRNA levels of integrin αv are at least 10 times higher than those of integrin β3 (data not shown). Thus, our results showing that knockdown of integrin β3 reduced both basal and activin B-induced cell migration, invasion and adhesion to vitronectin likely indicate that these effects are mediated by integrin αvβ3. Integrin αvβ3 is expressed in many types of cancer where it plays important roles in promoting angiogenesis and cancer cell adhesion, migration and invasion [36-42]. Though studies have shown that integrin αvβ3 is expressed in endometrioid and serous endometrial cancers [43, 44], its clinical and/or therapeutic relevance in these tumors has yet to be defined. Molecular therapies targeting αvβ3 have achieved positive results such as disease stabilization in advanced solid tumors [45, 46], as well as extended survival in high grade glioma [47, 48]. Interestingly, primary serous endometrial cancer cell migration and adhesion to vitronectin were reduced following treatment with inhibitory anti-integrin αv antibody [44]. However, endometrial cancers have also been shown to express integrin αvβ5 and αvβ6 [44, 49], though their functional roles are unknown. Regardless, our study demonstrates that integrin β3 (likely αvβ3) may constitute a novel therapeutic target in type II endometrial cancers by virtue of its ability to promote basal and activin B-induced cell adhesion, migration and invasion.

Apart from integrin-mediated cell-matrix contact, the metastatic capacity of tumors is also governed by cadherin mediated cell-cell adhesion [50]. In particular, cancer cell metastasis is often associated with epithelial-mesenchymal transition which is characterized by the down-regulation of E-cadherin and up-regulation of N-cadherin [51]. Increasing evidence suggests that endometrial cancers display a number of features associated with the epithelial-mesenchymal transition process [52]. Interestingly, we have recently demonstrated that activin B can stimulate human trophoblast cell invasion by up-regulating N-cadherin expression [30]. Whether modulation of E-cadherin or N-cadherin contributes to activin B-induced cell migration and invasion in endometrial cancer remains unknown and warrants further investigation.

Previous studies have shown that the expression of integrin β3 in endometrial cancer cells can be regulated by progesterone [53], macrophage migration inhibitory factor [54], and gonadotropin-releasing hormone [55]. Similar to our results for activin B in endometrial cancer cells, TGF-β1 has been shown to up-regulate integrin β3 in glioma cells [56], lung fibroblasts [57], and breast cancer cells [58], whereas it down-regulated integrin β3 in lymphoma cells [59]. Interestingly, our results show that while activin B activated both SMAD2 and SMAD3 in HEC-1B cells, only SMAD2 was activated in KLE cells. At present, we do not know why activin B failed to increase the levels of phosphorylated SMAD3 in KLE cells. However, it has previously been reported that TGF-β isoforms can induce SMAD3 phosphorylation in KLE cells [60], thus it does not appear to be a general defect in SMAD3 phosphorylation. Interestingly, depletion of SMAD3 abolished the stimulatory effects of activin B on integrin β3 expression in KLE cells, suggesting that SMAD3 phosphorylation is not required or endogenous levels of SMAD3 phosphorylation are sufficient for activin B-induced integrin β3 expression. Regardless, our siRNA results demonstrate that neither SMAD can compensate for the loss of the other, suggesting both SMAD2 and SMAD3 are required for the up-regulation of integrin β3 by activin B. Interestingly, knockdown of SMAD3 in lung fibroblasts did not alter TGF-β1-induced integrin β3 production, whereas it attenuated the up-regulation of integrin β5 [57]. Instead, c-Src and p38 MAPK signaling were required for the up-regulation of integrin β3 by TGF-β1 [57]. Future studies will be required to clarify how SMAD-dependent and -independent signaling is integrated at the level of the *ITGB3* promoter.

In summary, our results show that activin B stimulates the migration, invasion and adhesion of type II endometrial cancer cells. Moreover, these effects are mediated by the up-regulation of integrin β3 production in a SMAD2/3-SMAD4-dependent manner. Our study identifies novel molecular mechanisms that may contribute to the invasion and/or metastasis of type II endometrial cancers.

## MATERIALS AND METHODS

### Cell culture

HEC1B and KLE type II human endometrial cancer cell lines were purchased from the American Type Culture Collection (Manassas, VA) [61]. HEC-1B cells were cultured in Minimal Essential Medium (Gibco, Life Technologies, Burlington, ON) supplemented with 10% fetal bovine serum (FBS; Hyclone Laboratories Inc., Logan, UT). KLE cells were cultured in Dulbecco's Modified Eagle Medium/Nutrient Mixture F-12 (Gibco, Life Technologies) supplemented with 10% fetal bovine serum (FBS; Hyclone Laboratories Inc.). Cultures were maintained at 37°C in a humidified atmosphere of 5% CO2 in air.

### Antibodies and reagents

Rabbit polyclonal anti-human SMAD4 (#9515) antibody was obtained from Cell Signaling Technology. The rabbit monoclonal antibodies used in this study were: human phospho-SMAD2 (Ser465/467; 138D4, Cell Signaling Technology), human phospho-SMAD3 (Ser423/425; C25A9, Cell Signaling Technology), and human SMAD3 (C67H9, Cell Signaling Technology). The mouse monoclonal antibodies used were: human SMAD2 (L16D3, Cell Signaling Technology), human integrin β3 (#611141, BD Biosciences), human integrin αv (#611012, BD) and sea urchin α-tubulin (B-5-1-2, Santa Cruz). Horseradish peroxidase-conjugated goat anti-mouse IgG and goat anti-rabbit IgG were obtained from Bio-Rad Laboratories (Hercules, CA). SB431542 was purchased from Sigma-Aldrich (Oakville, ON). Recombinant human activin B was obtained from R&D Systems (Minneapolis, MN).

### MTT assay

MTT (3-(4,5-Dimethylthiazol-2-yl)-2,5-diphenyltetrazolium bromide; Sigma) assay was used to determine cell viability. Cells were seeded one day prior to treatment in 24-well plates (1×10^4^/well) with 500 μL of medium and then treated with activin B every 24 h for up to 72 h. MTT (final concentration of 0.5 mg/mL) was added at each time point and incubated for 4 h prior to removing the medium and adding DMSO to dissolve the crystals. Absorbances were measured at 490 nm using a microplate spectrophotometer.

### Transwell migration and invasion assays

Migration and invasion assays were performed in Boyden chambers with minor modifications [62]. Cell culture inserts (24-well, pore size 8 μm; BD Biosciences, Mississauga, ON) were seeded with 1×10^5^ cells in 250 μL of medium with 0.1% FBS. Un-coated inserts were used for migration assays whereas inserts pre-coated with growth factor reduced Matrigel (40 μL, 1 mg/mL; BD Biosciences) were used for invasion assays. Medium with 10% FBS (750 μL) was added to the lower chamber and served as a chemotactic agent. After incubation for 24 h (migration) or 48 h (invasion), non-migrating/invading cells were wiped from the upper side of the membrane and cells on the lower side were fixed in cold methanol and air dried. Cell nuclei were stained with Hoechst 33258 and counted using a Zeiss Axiophot epifluorescent microscope equipped with a digital camera (QImaging, Surrey, BC). Each individual experiment had triplicate inserts and five microscopic fields (obtained from middle, upper, lower, right and left parts of membrane) were counted per insert using Northern Eclipse 6.0 software.

### Adhesion assays

96-well plates were coated overnight at 4°C with vitronectin (1 μg/cm^2^; R&D Systems), fibronectin (10 μg/cm^2^; R&D Systems), Matrigel (5 μg/cm^2^; BD Biosicences) or collagen IV (10 μg/cm^2^; R&D Systems) and then blocked for 1 h with 0.5% bovine serum albumin. Cells were seeded at a density of 4 × 10^4^ cells/well and incubated at 37°C for 1.5 h. Non-adherent cells were removed by washing with PBS, and adherent cells were fixed with cold methanol and stained with 0.1% crystal violet for 25 min at room temperature. After removing the crystal violet solution, the stained cells were washed with water and 10% acetic acid was added to dissolve the crystal violet. Absorbances were measured at 590 nm using a microplate spectrophotometer.

### Reverse transcription quantitative real-time PCR (RT-qPCR)

Total RNA was extracted using TRIzol reagent (Invitrogen, Life Technologies, Burlington, ON) in accordance with the manufacturer's instructions. Reverse transcription was performed with 2 μg RNA, random primers and M-MLV reverse transcriptase (Promega, Madison, WI). The primers used for SYBR Green RT-qPCR were: integrin β3, 5′-GAA GGC TGG CAG GCA TTG-3′ (forward) and 5′-AAT GAT TGT CAC TAC CAA CAT GAC ACT-3′ (reverse); integrin αv, 5′-TGC CCA GCG CGT CTT C-3′ (forward) and 5′-TGG GTG GTG TTT GCT TTG G-3′ (reverse); SMAD2, 5′-GCC TTT ACA GCT TCT CTG AAC AA-3′ (forward) and 5′-ATG TGG CAA TCC TTT TCG AT-3′ (reverse); SMAD3, 5′-CCC CAG CAC ATA ATA ACT TGG-3′ (forward) and 5′-AGG AGA TGG AGC ACC AGA AG-3′ (reverse); SMAD4, 5′-TGG CCC AGG ATC AGT AGG T-3′ (forward) and 5′-CAT CAA CAC CAA TTC CAG CA-3′ (reverse) and GAPDH, 5′-GAG TCA ACG GAT TTG GTC GT-3′ (forward) and 5′- GAC AAG CTT CCC GTT CTC AG-3′ (reverse). RT-qPCR was performed using an Applied Biosystems 7300 Real-Time PCR System equipped with 96-well optical reaction plates. The specificity of each assay was validated by melting curve analysis and agarose gel electrophoresis of the PCR products. Assay performance was validated by assessing amplification efficiencies by means of calibration curves, and ensuring that the plot of log input amount versus ΔCq has a slope < |0.1|. At least three separate experiments were performed and each sample was assayed in triplicate. A mean value of the triplicates was used for the determination of relative mRNA levels by the comparative Cq method with GAPDH as the reference gene and using the formula 2^−ΔΔCq^.

### Western blot

Cells were lysed in ice cold lysis buffer (Cell Signaling Technology) with added protease inhibitor cocktail (Sigma-Aldrich). Extracts were centrifuged at 20,000×g for 10 min at 4°C and supernatant protein concentrations were determined using the DC Protein Assay (Bio-Rad Laboratories). Equal amounts of protein (50 μg) were separated by SDS polyacrylamide gel electrophoresis and transferred onto PVDF membranes. After blocking for 1 h with 5% non-fat dry milk in Tris-buffered saline (TBS), the membranes were incubated overnight at 4°C with primary antibodies that were diluted 1000-fold in 5% non-fat milk-TBS. Following primary antibody incubation, the membranes were incubated with the appropriate HRP-conjugated secondary antibody. Immunoreactive bands were detected using enhanced chemiluminescent substrate or SuperSignal West Femto chemiluminescent substrate (Thermo Fisher) and X-ray film. Membranes were stripped with stripping buffer (50 mM Tris-HCl pH 7.6, 10 mM β-mercaptoethanol, and 1% SDS) at 50°C for 30 min and reprobed with anti-α-tubulin antibody. Densitometric quantification was performed using Scion Image software (Scion Corp, Frederick, MD) with α-tubulin as the internal control for normalization.

### Small interfering RNA (siRNA) transfection

To knock down endogenous integrin β3, SMAD2, SMAD3 and SMAD4, forty percent confluent cells were transfected for 48 h with 20 nM ON-TARGET*plus* SMART pool siRNA targeting human integrin β3, SMAD2, SMAD3 and SMAD4 (Dharmacon, Lafayette, CO) using Lipofectamine RNAiMAX (Invitrogen, Life Technologies). ON-TARGET*plus* Non-targeting pool siRNA (Dharmacon) was used as the control.

### Statistical analysis

Results are presented as the mean ± SEM of at least three independent experiments. For experiments involving only two groups, results were analyzed by Two-Sample *t*-test assuming unequal variances using Excel. Multiple group comparisons were analyzed by one-way ANOVA followed by Student-Newman-Keuls test using PRISM software (GraphPad Software). Means were considered significantly different if *P* < 0.05 and are indicated by different letters.

## SUPPLEMENTARY MATERIAL FIGURE


