# Supplementary material for: Activin B induces human endometrial cancer cell adhesion, migration and invasion by up-regulating integrin β3 via SMAD2/3 signaling
Source: Oncotarget. 2015 Aug 28;6(31):31659–73. doi: 10.18632/oncotarget.5229 (PMC4741631; doi:10.18632/oncotarget.5229)
Supplement: Supplementary file 1 [file oncotarget-06-31659-s001.pdf]

# Activin B induces human endometrial cancer cell adhesion, migration and invasion by up-regulating integrin β3 via SMAD2/3 signaling

**Supplementary Material**

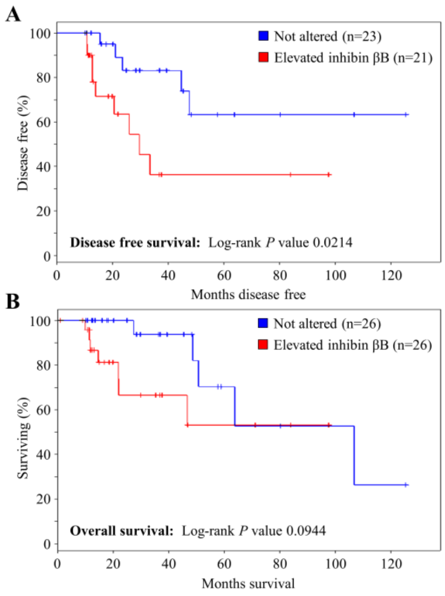


**Supplementary Figure 1.** Elevated inhibin βB is associated with reduced disease free survival and a trend towards reduced overall survival in serous endometrial cancers. The cBioPortal for Cancer Genomics was used to query endometrial carcinomas with serous histology from The Cancer Genome Atlas (n=53) for up-regulation of inhibin βB subunit mRNA above the median. Disease free (A) and overall (B) survival differences between unaltered samples and those with elevated inhibin βB are displayed as Kaplan-Meier survival curves with a *P* value from a Log-rank test.
